# Useful island block geometries of a passive intensity modulator used for intensity‐modulated bolus electron conformal therapy

**DOI:** 10.1002/acm2.13079

**Published:** 2020-11-18

**Authors:** Erin L. Chambers, Robert L. Carver, Kenneth R. Hogstrom

**Affiliations:** ^1^ Department of Physics and Astronomy Louisiana State University Baton Rouge LA USA; ^2^ Mary Bird Perkins Cancer Center Baton Rouge LA USA

**Keywords:** electron therapy, intensity modulation, bolus, conformal therapy

## Abstract

**Purpose:**

This project determined the range of island block geometric configurations useful for the clinical utilization of intensity‐modulated bolus electron conformal therapy (IM‐BECT).

**Methods:**

Multiple half‐beam island block geometries were studied for seven electron energies 7‐20 MeV at 100 and 103 cm source‐to‐surface distance (SSD). We studied relative fluence distributions at 0.5 cm and 2.0 cm depths in water, resulting in 28 unique beam conditions. For each beam condition, we studied intensity reduction factor (IRF) values of 0.70, 0.75, 0.80, 0.85, 0.90, and 0.95, and hexagonal packing separations for the island blocks of 0.50, 0.75, 1.00, 1.25, and 1.50 cm, that is, 30 unique IM configurations and 840 unique beam‐IM combinations. A combination was deemed acceptable if the average intensity downstream of the intensity modulator agreed within 2% of that intended and the variation in fluence was less than ±2%.

**Results:**

For 100 cm SSD, and for 0.5 cm depth, results showed that beam energies above 13 MeV did not exhibit sufficient scatter to produce clinically acceptable fluence (intensity) distributions for all IRF values (0.70–0.95). In particular, 20 MeV fluence distributions were unacceptable for any values, and acceptable 16 MeV fluence distributions were limited to a minimum IRF of 0.85. For the 2.0 cm depth, beam energies up to and including 20 MeV had acceptable fluence distributions. For 103 cm SSD and for 0.5 cm and 2.0 cm depths, results showed that all beam energies (7–20 MeV) had clinically acceptable fluence distributions for all IRF values (0.70–0.95). In general, the more clinically likely 103 cm SSD had acceptable fluence distributions with larger separations (r), which allow larger block diameters.

**Conclusion:**

The geometric operating range of island block separations and IRF values (block diameters) producing clinically appropriate IM electron beams has been determined.

## 1 INTRODUCTION

Electron beam therapy has been a standard modality in radiation treatment for over 60 years. Electron beams with energies between 6 and 20 MeV (R_90_ = 1.8–6.0 cm) are characterized by high surface dose, relatively uniform dose plateau, sharp distal dose fall off, and low exit x‐ray dose. These characteristics have allowed superficial cancers within 6 cm of surface to be treated while minimizing dose to underlying critical structures.^1^ Historically, electrons have been the modality of choice for (1) the treatment of skin, lip, and head and neck tumors, (2) boost doses to superficial lymph nodes, and (3) post‐mastectomy chest wall irradiation.^2^, ^3^, ^4^, ^5^ Electron therapy planning often utilizes a single beam of energy just sufficient for R_90_ of the electron beam to exceed maximum planning target volume (PTV) depth. However, delivery is often complicated by internal heterogeneities, irregular patient surface, and variable depth of the distal PTV surface resulting in needless overdosing of distal structures, in which case some form of electron conformal therapy (ECT) is desirable.^6^

**Fig. 1:**
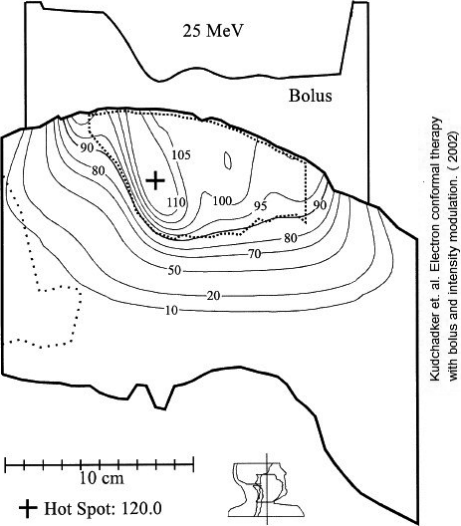
Isodosedistribution(100% = given dose) for a 25 MeV bolus ECT treatment plan of a right buccal mucosa patient; 90% dose surface conforms to the distal surface of the planning target volume (dotted line). From Kudchadker et al (2003).

The goals of electron conformal therapy are to conform the distal 90% dose surface to the distal surface of the PTV, provide a homogeneous or prescribed heterogeneous dose to the PTV, and maximize dose sparing of critical structures deep to the PTV.^6^ One of three methods described by Hogstrom et al.^6^ is Bolus ECT, which uses a single energy electron beam to deliver a dose distribution that conforms the 90% dose surface to the distal surface of the PTV. This is accomplished by using variable‐thickness bolus, a nearly water‐equivalent material which is placed on the patient surface. Algorithms for bolus design were first created by Low et al.^7^ and later by Su et al^8^; these algorithms projected ray lines from the electron virtual source to the distal margin of the PTV and applied a succession of bolus operators to generate a bolus structure. Fig. 1 illustrates how bolus ECT conforms the 90% dose surface to the distal PTV surface of a right buccal mucosa patient. Boluses have been readily available to clinics since the introduction in 2009 of bolus design software (BolusECT®) in the p.d software system and bolus fabrication using milling technology, available from.decimal, LLC (Sanford, FL, http://dotdecimal.com/products/electrons/bolusect/). BolusECT® utilizes design operators based on Low et al.^7^ and a pencil beam redefinition algorithm for dose calculations,^9^, ^10^, ^11^, ^12^ which has been verified for patient‐like volumes.

Bolus ECT has been used for multiple sites, which include posterior chest wall^8^, ^13^, ^14^; post‐mastectomy chest wall^14^, ^15^, ^16^, ^17^; ear, parotid, and buccal mucosa,^14^, ^18^ nose,^19^ and extremities (hand and foot).^8^ The irregular upstream bolus surface can often cause undesirable dose heterogeneities in the PTV. However, it was shown by Kudchadker et al.^14^ that the introduction of modest intensity modulation (70%‐100%) across the beam can significantly reduce PTV heterogeneity for some patients.

**Fig. 2:**
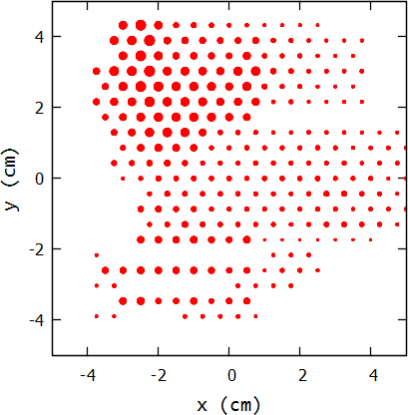
Beam’s eye view of intensity modulator design for the buccal mucosa patient shown in Fig.1. Red circles show variable circular cross sections of island blocks.

Initially, delivery of intensity modulation was envisioned using eMLCs, such as those reported by Hogstrom et al.^20^ and Gauer et al.^21^; however, access to said devices by the typical clinic has not been forthcoming. As an alternative, Hogstrom et al.^22^ reported a passive method for electron intensity modulation, which consists of a matrix of variable small‐diameter, high‐density island blocks, as shown in Fig. 2. The matrix consists of small diameter, tungsten cylinders (eg 0.2 to 0.6 cm diameter x 0.6 cm thick) of varying diameter placed on a hexagonal grid with approximately 0.6 cm spacing. The cylinders, referred to as island blocks and whose axes follow diverging fan lines, are embedded in a low density machineable foam (≈0.1 g*cm^‐3^) that fits inside the aperture of the electron beam cutout located in the bottom of the electron applicator. The matrix can closely produce the desired intensity modulation while the beam energy remains almost unchanged (≈0.2 MeV decrease due to the foam substrate on which island blocks are mounted^23^). The local beam intensity is determined by the fraction of the beam locally removed by the island blocks. Because of the multiple Coulomb scattering (MCS) of the electron beam, fluence is filled in behind the blocks 5–10 cm downstream. Using small diameter islands blocks, intensity modulators can be designed for suitable intensity‐modulated bolus electron conformal therapy (IM‐BECT).

The purpose of this study was to investigate how best to deliver passive intensity modulation for IM‐BECT, where intensity modulation in the range of 70%–100% should be sufficient. Intensity modulation using island blocks can be achieved by either modifying the diameter and/or spacing of the island blocks. Our study only investigates island blocks placed on a hexagonal grid and then varying their diameters to achieve the appropriate IM, as illustrated in Fig. 2.

The fraction of area locally blocked, which controls the intensity reduction factor (IRF) locally, is governed by the ratio of block diameter (d) to hexagonal grid spacing (r),^22^

(1) $$\mathrm{IRF}=1.0‐\frac{\pi }{2\sqrt{3}}{\left(\frac{d}{r}\right)}^{2}.$$

Hence, there are an infinite number of potential configurations for a single d/r ratio. Making d too small has the undesired effects of (1) requiring too many island blocks, likely increasing manufacturing costs, and (2) increasing the number of electrons scattering into and out of the blocks, which degrade the fluence distribution. Making d too large has the undesirable effect of not allowing sufficient electrons, via MCS, to fill in fluence behind the blocks, which causes hot and cold spots. Therefore, to achieve how best to deliver passive intensity modulation for IM‐BECT, this study determined near‐optimal values of island block separation (r) for patient‐specific conditions, that is, beam energy, source‐to‐surface distance (SSD), depth in water, and range of IRF values.

Using a pencil beam algorithm for electron fluence calculations and assuming perfect collimation (ie, ignoring MCS into and out of the island blocks and bremsstrahlung in the island blocks), we determined island block geometries (diameters and separations) for IRF values in the range of 70–95%, electron beams from 7 to 20 MeV (R_90_ =2.0, 2.5, 3.0, 3.5, 4.0, 5.0, and 6.0 cm), 100 and 103 cm SSD, and depths in water of 0.5 and 2.0 cm. Perfect collimation can be assumed because MCS into and from the island blocks and bremsstrahlung attenuation and production in the island blocks have only a small effect on the underlying dose distribution^23^, which has insignificant effect on selecting the near‐optimal values of island block separation (r). Its impact on the underlying dose distribution, which can be a few percent^23^, is otherwise important and will be reported in subsequent studies using Monte Carlo (MC) dose calculations to provide data for modifications to the PBRA, currently used for IM‐BECT planning.

## 2 METHODS

### 2.1 Conditions of study

Multiple island block geometries were studied for seven electron energies 7, 9, 10, 11, 13, 16, and 20 MeV. 100 cm and 103 cm SSD was selected, as bolus ECT patients are typically treated at 105 cm source‐to‐skin surface distance (SSD_skin_) to ensure the bolus does not collide with the applicator, which extends to 95 cm source‐to‐collimator distance (SCD). Bolus thickness typically averages approximately 2.0 cm, so that the SSD to the bolus surface is approximately 103 cm. The 100 cm SSD was selected as an upper limit where either the patient surface is closer to the source and/or a bolus thicker than 2.0 cm is required. Beam intensity modulation is intended to be reflected in the patient; therefore, we elected to study relative fluence (intensity) at depths of 0.5 cm and 2.0 cm in water. This resulted in 28 unique beam conditions studied (7 energies x 2 SSDs x 2 depths).

Based on Kudchadker et al,^14^ we studied IRF values of 0.70, 0.75, 0.80, 0.85, 0.90, and 0.95. We studied potentially practical hexagonal separations for the island blocks of 0.50, 0.75, 1.00, 1.25, and 1.50 cm. Once a specific IRF and r are selected, d is determined using Eq. (1). These values, independent of beam conditions, are listed in Table 1 for 30 different IM configurations (6 IRF values x 5 island block separations). Hence, relative fluence (intensity) distributions were calculated for 840 unique combinations (30 IM configurations × 28 beam conditions).

**Table 1:** Island block diameters (d) in cm for select combinations of intensity reduction factors (IRF) and hexagonal island block separations (r) calculated using Equation 1.

IRF	r = 0.5 cm	r = 0.75 cm	r = 1.0 cm	r = 1.25 cm	r = 1.5 cm
0.95	0.117	0.176	0.235	0.294	0.352
0.90	0.166	0.249	0.332	0.415	0.498
0.85	0.203	0.305	0.407	0.508	0.610
0.80	0.235	0.352	0.470	0.587	0.704
0.75	0.263	0.394	0.525	0.656	0.788
0.70	0.288	0.431	0.575	0.719	0.863

### 2.2 Calculation of relative fluence distributions

**Fig. 3:**
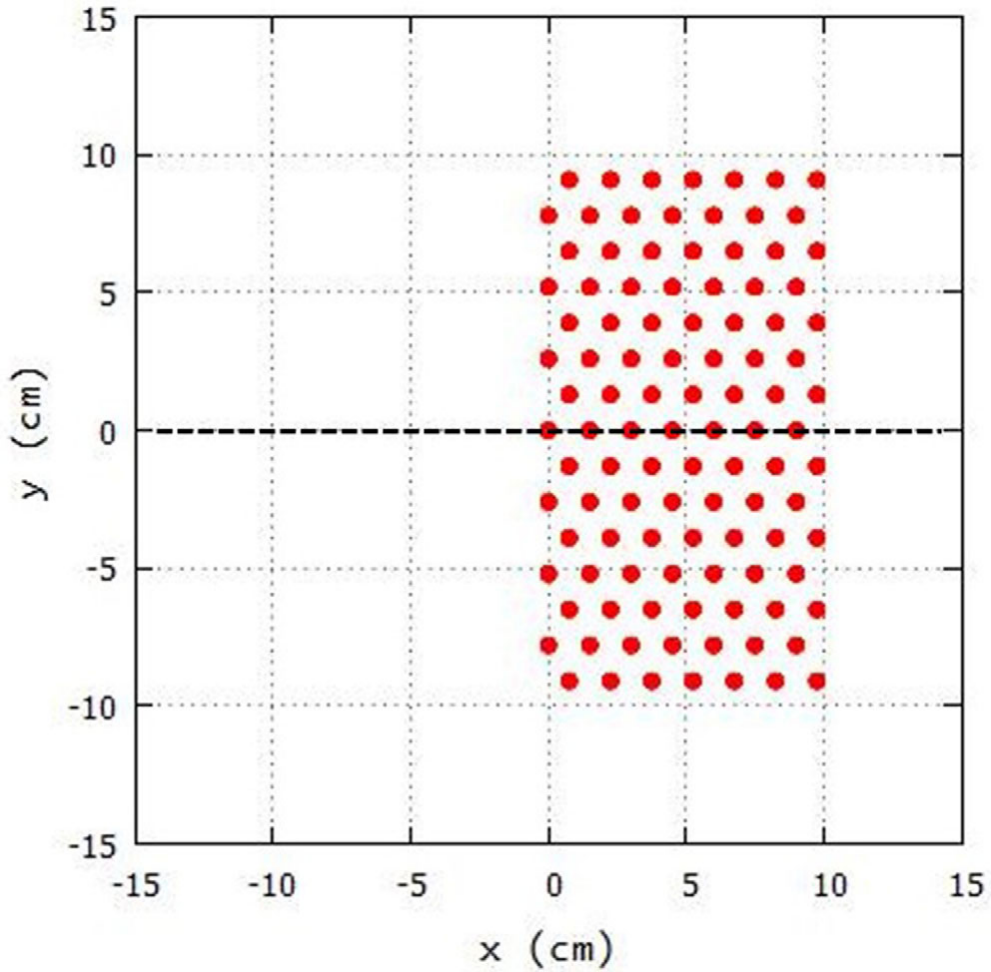
Sampleislandblock matrix used to determine acceptable (r,d) combinations. For this example, blocks with (r,d) = (1.50 cm, 0.498 cm), having an IRF = 0.90, are hexagonally packed in the + x half plane of the 20 × 20 cm^2^field at the positions indicated by the red circles. Red circles show circular cross sections of island blocks. Dashed line at y=0 indicates the xz plane in which off‐axis profiles of relative intensity versus x were calculated at depths of z = 0.5 and 2.0 cm in water.

Relative fluence was calculated in a plane perpendicular to central axis using the pencil beam algorithm (PBA) following the convention of Hogstrom et al,^24^ assuming perfect collimation. The circular cross‐sections of the cylindrical island blocks were modeled as small square cross‐sections of equal area. This calculation, detailed by Chambers,^25^ was used to determine which block geometries (r and d) produce intensity distributions (70–95%) acceptable for clinical use. Calculations were performed for monoenergetic beams of energies 7, 9, 10, 11, 13, 16, and 20 MeV with $\sigma_{\theta_{x}}$ values of 0.069, 0.055, 0.050, 0.045, 0.040, 0.033, and 0.027 radians, respectively, using a 20 × 20 cm^2^ field at depths of 0.5 cm and 2.0 cm. The collimating plane to water surface distances (air gaps) were taken to be 5 cm and 8 cm, corresponding to the 100 cm and 103 cm SSD, respectively, for a clinically divergent beam. Relative fluence profiles were calculated at the two depths in water for a 20 × 20 cm^2^ field, which was half‐covered by identical cylindrical island blocks placed in a hexagonal array with separation r and block diameter d. A schematic of one such block matrix is shown in Fig. 3. Plots of relative fluence vs x position at y = 0 were generated. Each intensity modulator was assumed to be located in the 20 × 20 cm^2^ block aperture, that is, 95 cm SCD.

### 2.3 Evaluation metrics

**Fig. 4:**
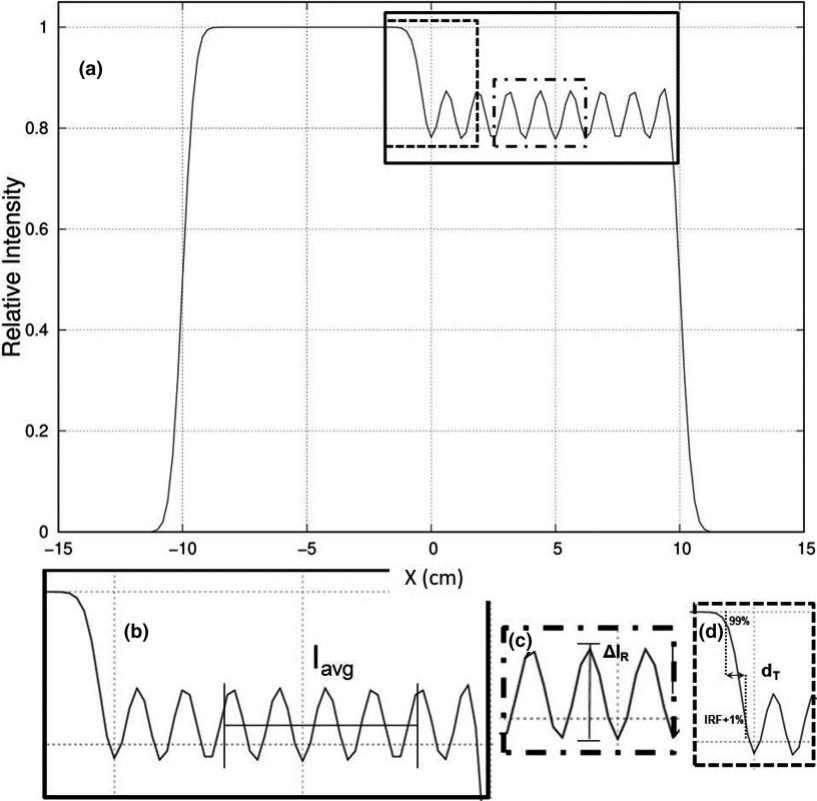
Illustrationsofthe assessment metrics. (a) Relative intensity vs. x‐position profile (y = 0 cm) for E = 13 MeV, FS = 20 × 20 cm^2^,source‐to‐surface distance (SSD) = 100 cm, depth z = 2.0 cm is plotted for a matrix of island blocks at locations shown in Fig.3, with island blocks parameters of r = 1.25 cm and d = 0.415 cm (IRF = 0.85). (b) I_avg_is the average intensity in the specified region. (c) ∆I_R_is the maximum spread in the specified region. (d) Distance of transition (d_T_) is the distance from the 99% to the (IRF‐1%) relative intensity.

Calculated relative fluence (intensity) distributions downstream of the half‐beam intensity modulators were evaluated using two metrics: average blocked intensity (I_avg_) and ripple intensity (∆I_R_). I_avg_ was the average intensity for |y| < 7.5 cm and 2.5 ≤ x ≤ 7.5 cm. ∆I_R_ was defined as the difference between the maximum and minimum intensities within the blocked region, defined by |y| < 7.5 cm and 2.5 ≤ x ≤ 7.5 cm. A block configuration (r,d) for each energy, was considered unacceptable if I_avg_ differed from the intended IRF by more than 2% or if ∆I_R_ was greater than 4%, which was always symmetric (± 2%) about I_avg_. Criteria for these first two metrics require all intensities to be within 4% of the intended intensity; however, as results will show, I_avg_ was always within 0.1%, so that all intensities would be within ± 2% of the intended intensity. Also, a third metric called distance of transition (d_T_) was calculated for all combinations. d_T_ was defined as the average straight‐line distance along the x‐axis from relative intensities 0.99 to IRF + 0.01 for each x profile (every 0.2 cm in y) such that |y| < 7.5 cm. This distance is a measure of the spatial resolution of the specific IRF, that is, the distance required to modify the intensity. Fig. 4 illustrates these three metrics.

## 3 RESULTS AND DISCUSSION

### 3.1 Intensity profiles and metrics

**Fig. 5:**
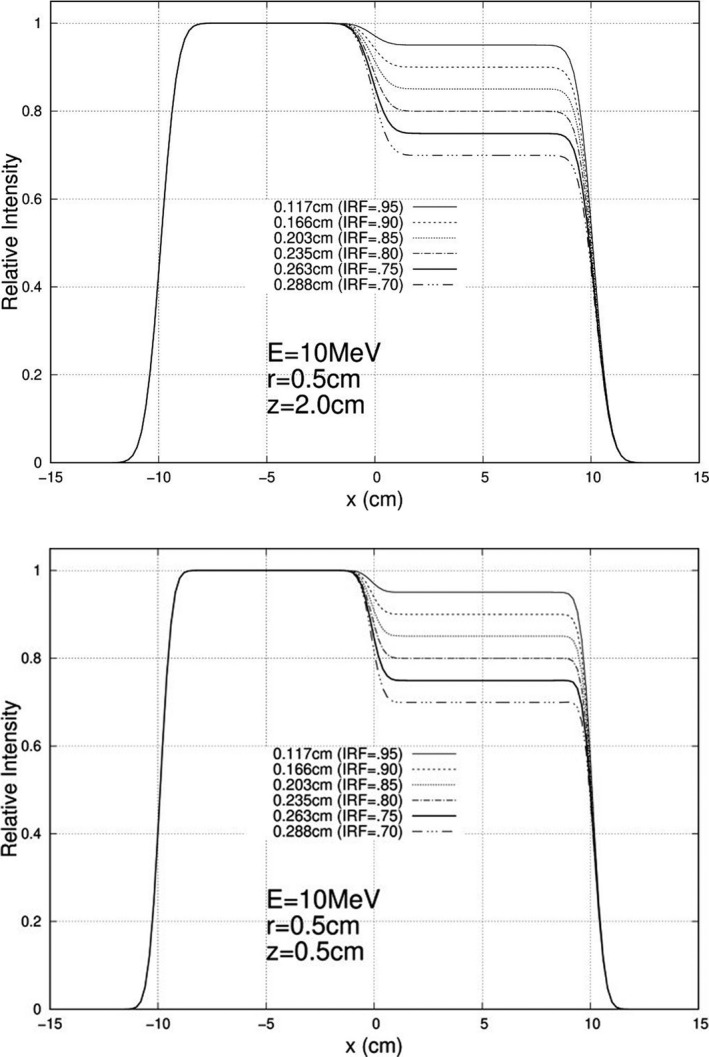
Profiles at y = 0 cm for 10 MeV, 20 × 20 cm^2^ half‐modulated field (r = 0.5 cm), 103 cm source‐to‐surface distance (SSD): depth z = 0.5 cm (top) and z = 2.0 cm (bottom). The computed island block diameters (d) for 0.70, 0.75, 0.80, 0.85, 0.90, and 0.95 IRF values are listed in each plot's inserted key.

**Fig. 6:**
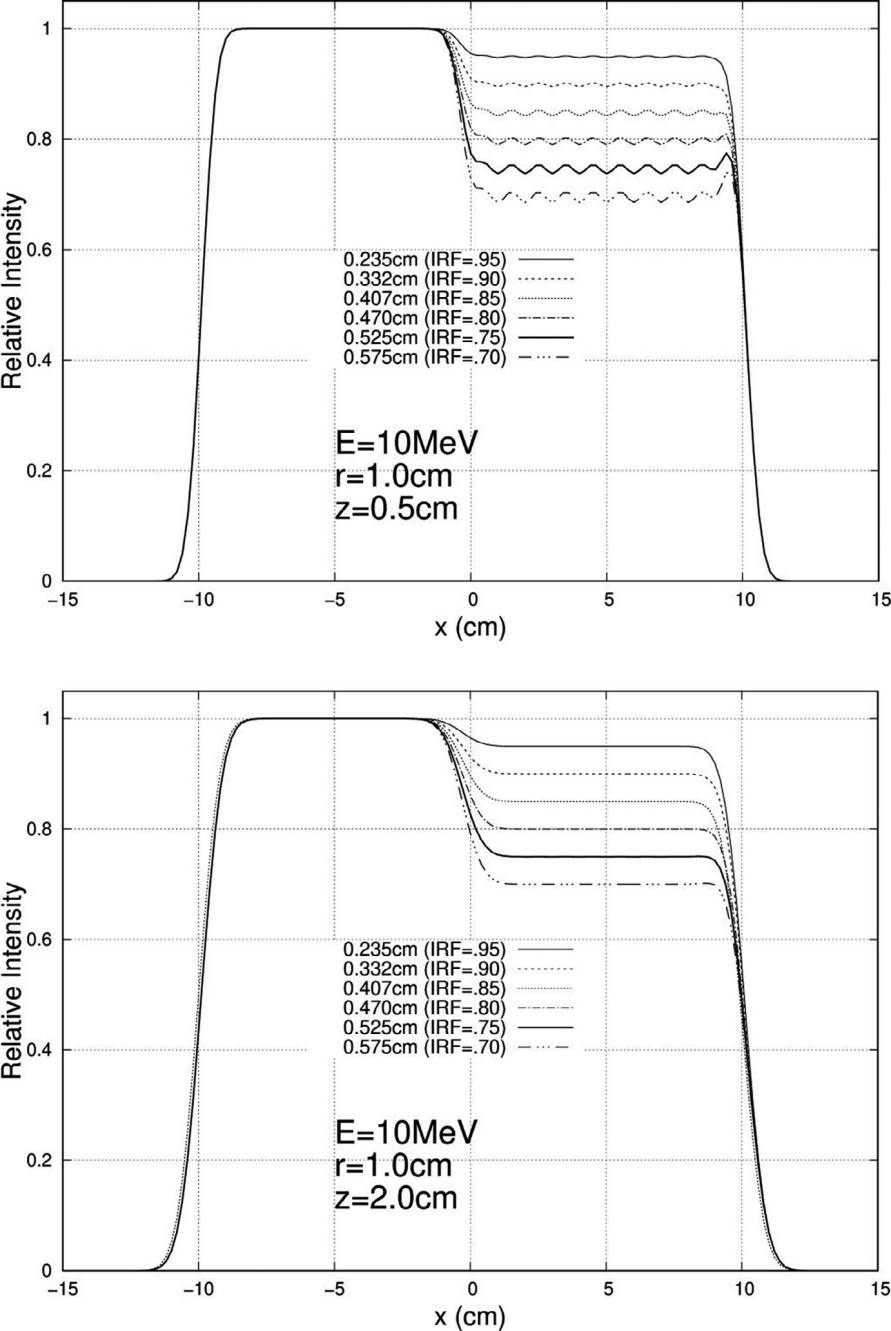
Profiles at y = 0 cm for 10 MeV, 20 × 20 cm^2^ half‐modulated field (r = 1.0 cm), 103 cm source‐to‐surface distance (SSD): depth z = 0.5 cm (top) and z = 2.0 cm (bottom). The computed island block diameters (d) for 0.70, 0.75, 0.80, 0.85, 0.90, and 0.95 IRF values are listed in each plot's inserted key.

**Fig. 7:**
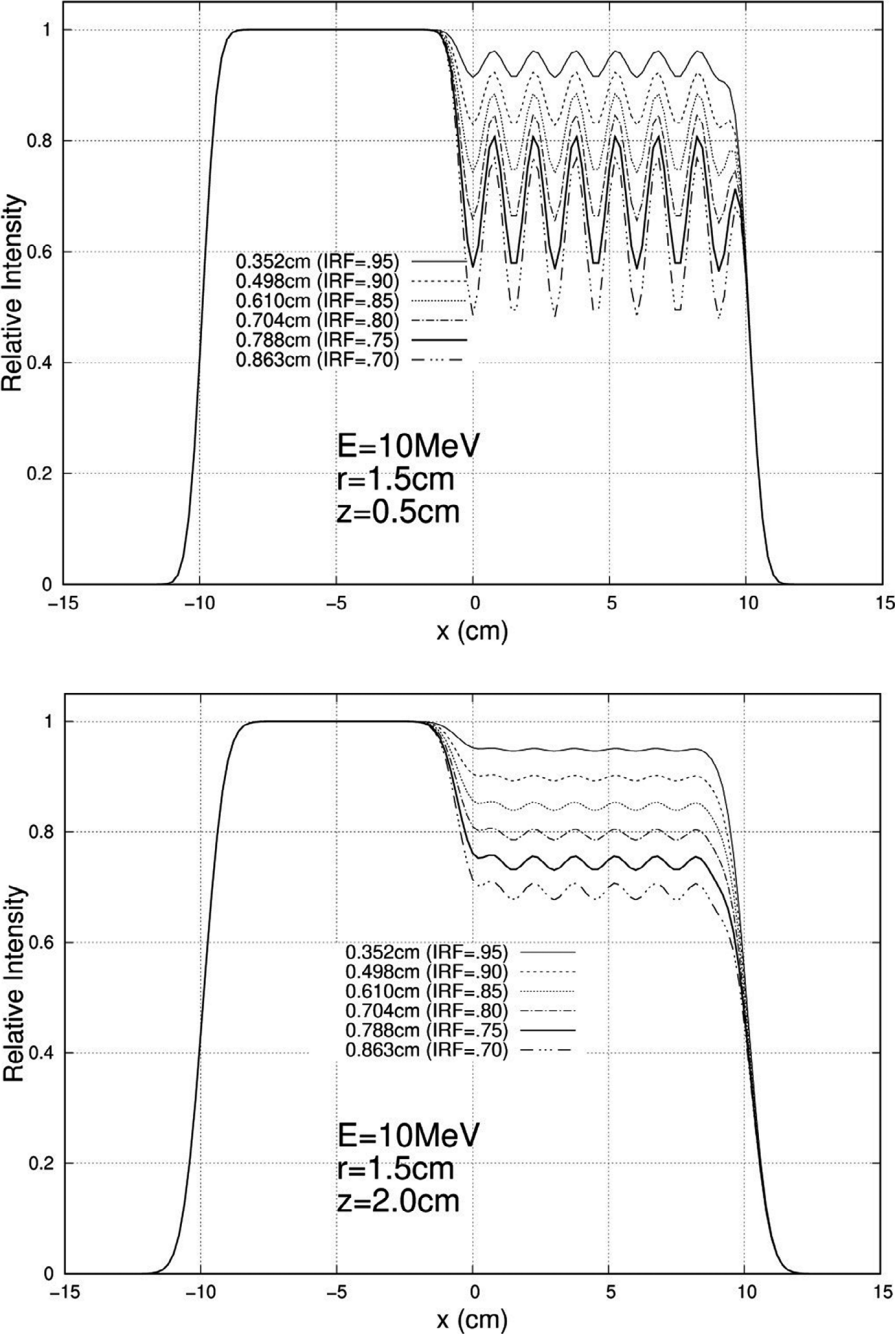
Profiles at y = 0 cm for 10 MeV, 20 × 20 cm^2^ half‐modulated field (r = 1.5 cm), 103 cm source‐to‐surface distance (SSD): depth z = 0.5 cm (top) and z = 2.0 cm (bottom). The computed island block diameters (d) for 0.70, 0.75, 0.80, 0.85, 0.90, and 0.95 IRF values are listed in each plot's inserted key.

**Fig. 8:**
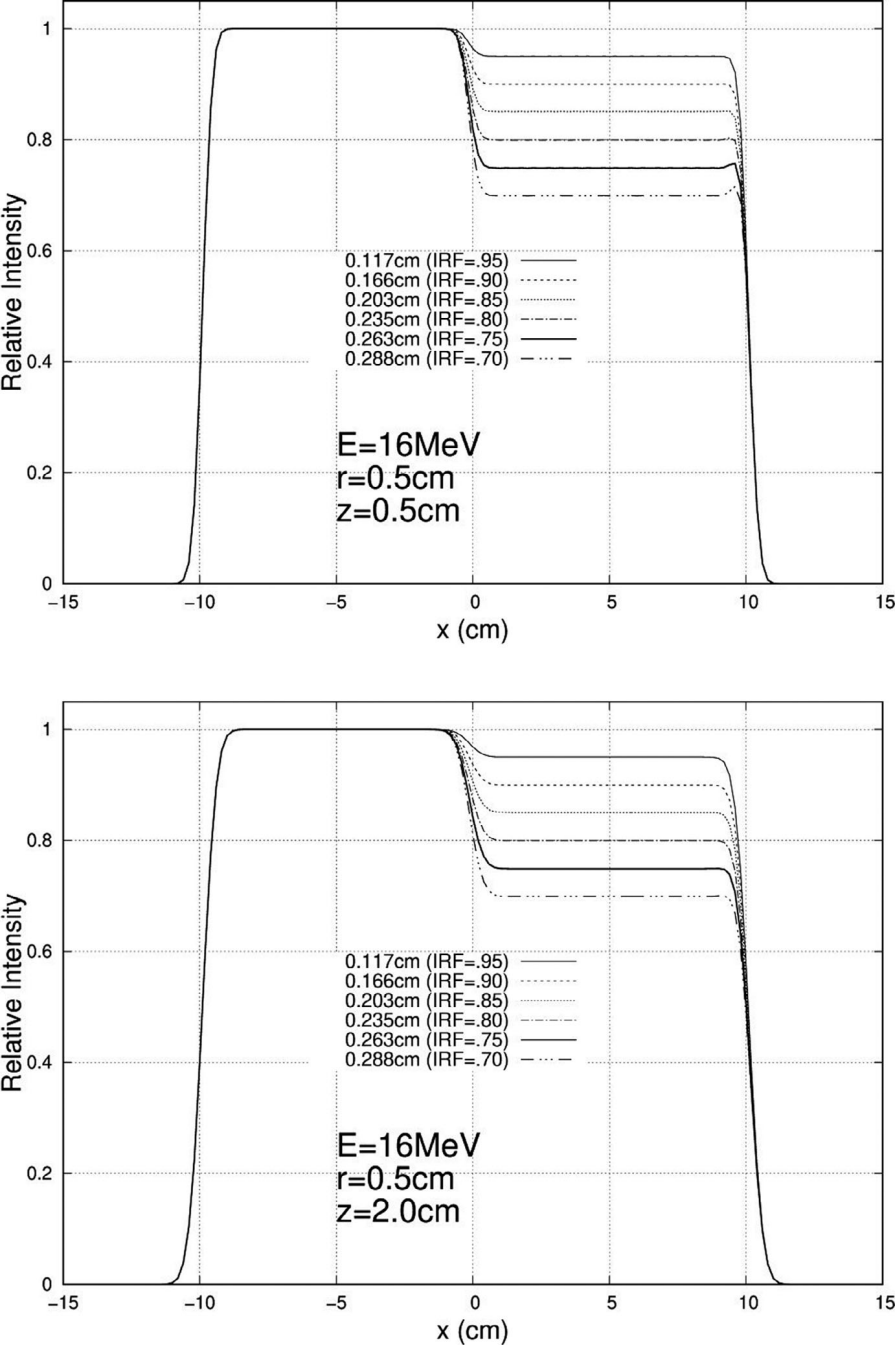
Profiles at y = 0 cm for 16 MeV, 20 × 20 cm^2^ half‐modulated field (r = 0.5 cm), 103 cm source‐to‐surface distance (SSD): depth z = 0.5 cm (top) and z = 2.0 cm (bottom). The computed island block diameters (d) for 0.70, 0.75, 0.80, 0.85, 0.90, and 0.95 IRF values are listed in each plot's inserted key.

**Fig. 9:**
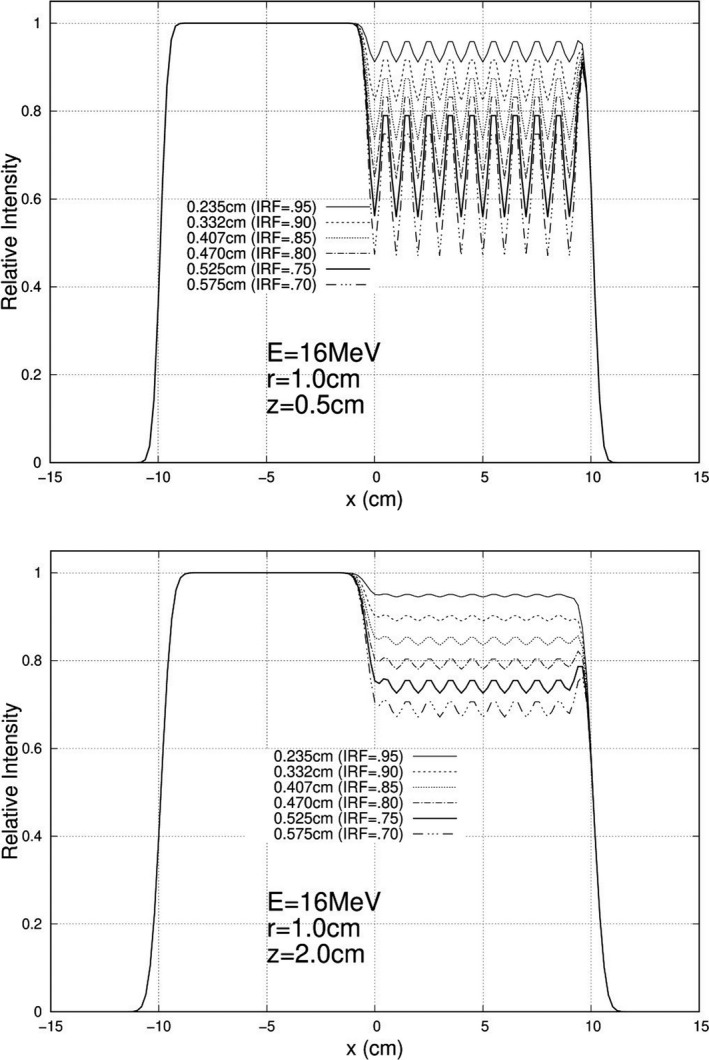
Profiles at y = 0 cm for 16 MeV, 20 × 20 cm^2^ half‐modulated field (r = 1.0 cm), 103 cm source‐to‐surface distance (SSD): depth z = 0.5 cm (top) and z = 2.0 cm (bottom). The computed island block diameters (d) for 0.70, 0.75, 0.80, 0.85, 0.90, and 0.95 IRF values are listed in each plot's inserted key.

**Fig. 10:**
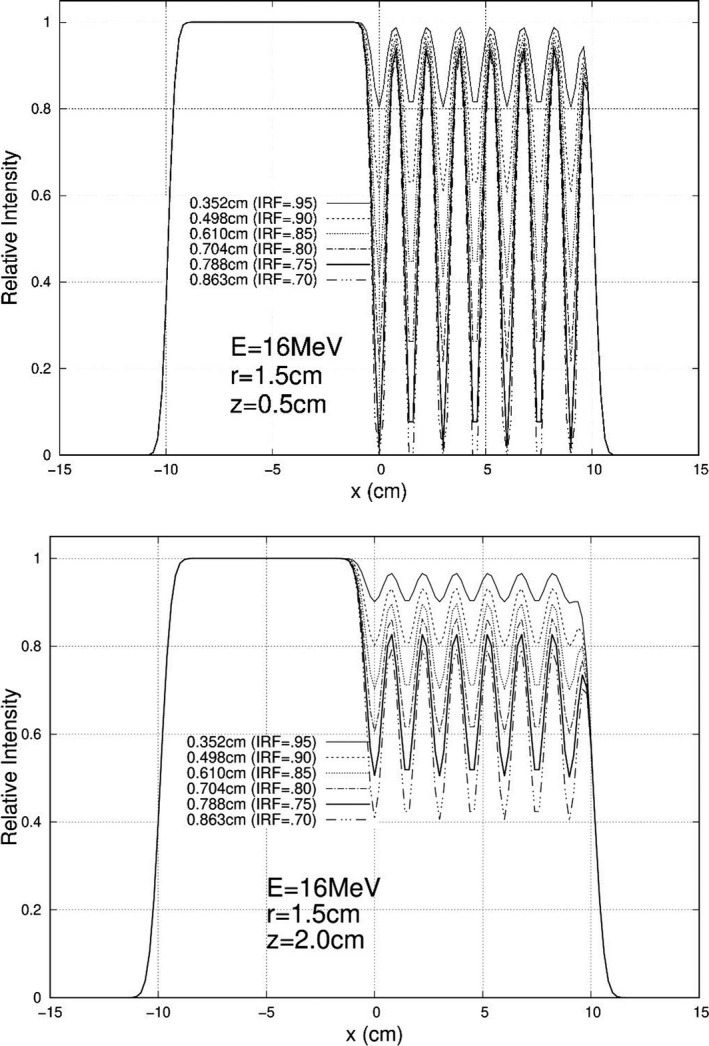
Profiles at y = 0 cm for 16 MeV, 20 × 20 cm^2^ half‐modulated field (r = 1.5 cm), 103 cm source‐to‐surface distance (SSD): depth z = 0.5 cm (top) and z = 2.0 cm (bottom). The computed island block diameters (d) for 0.70, 0.75, 0.80, 0.85, 0.90, and 0.95 IRF values are listed in each plot's inserted key.

Calculations were performed and plotted for each of the 840 unique combinations specified above, and exemplary results are plotted here. For 10 MeV, Figs. 5, 6, and 7 show relative intensity vs x profiles at y = 0 for hexagonal separations r = 0.5, 1.0, and 1.5 cm respectively for each IRF (0.70, 0.75, 0.80, 0.85, 0.90, and 0.95) at calculation depths (z) of 0.5 cm and 2.0 cm for 103 cm SSD. Similar results at 16 MeV are shown in Figs. 8, 9, and 10, respectively. The complete set of y = 0 profiles for all r (0.5, 0.75, 1.0, 1.25, and 1.5 cm) and IRF (0.70, 0.75, 0.80, 0.85, 0.90, and 0.95) combinations at all energies (7, 9, 10, 11, 13, 16, and 20 MeV), both SSDs (100 and 103 cm), and both depths (0.5 and 2.0 cm) is documented in Appendix B of Chamber’s thesis.^25^

These relative electron intensity x‐profiles were scored and evaluated using the three metrics I_avg_, ∆I_R_, and d_T_, as previously defined in Section 2.C. Tables 2 and 3 show the scoring values for the 10 MeV and 16 MeV distributions, respectively, at 103 cm SSD. Tables for all seven energies and two SSDs are documented in Appendix C of Chambers’ thesis.^25^

**Table 2:** Metrics summary for 10 MeV at 103 cm source‐to‐surface distance (SSD) at y = 0 cm 20 × 20 cm^2^ half‐modulated field for z = 0.5 cm (middle columns) and z = 2.0 cm (right columns).

r (cm)	IRF	d (cm)	z = 0.5 cm			z = 2.0 cm		
			d_T_	I_avg_	ΔI_R_	d_T_	I_avg_	ΔI_R_
0.5	0.95	0.117	0.63	0.950	0.000	1.05	0.950	0.000
	0.90	0.166	1.03	0.900	0.000	1.66	0.900	0.000
	0.85	0.203	1.42	0.851	0.000	1.87	0.851	0.000
	0.80	0.235	1.42	0.800	0.000	2.07	0.800	0.000
	0.75	0.263	1.43	0.749	0.000	2.26	0.749	0.000
	0.70	0.288	1.62	0.699	0.000	2.27	0.699	0.000
0.75	0.95	0.176	0.79	0.950	0.000	1.15	0.950	0.000
	0.90	0.249	1.16	0.900	0.000	1.67	0.900	0.000
	0.85	0.305	1.22	0.850	0.000	2.02	0.850	0.000
	0.80	0.352	1.42	0.800	0.000	2.07	0.800	0.000
	0.75	0.394	1.58	0.750	0.000	2.81	0.750	0.000
	0.70	0.431	1.62	0.701	0.001	2.44	0.701	0.000
1	0.95	0.235	0.67	0.950	0.004	1.04	0.950	0.000
	0.90	0.332	1.03	0.900	0.007	1.61	0.900	0.000
	0.85	0.407	1.24	0.850	0.011	1.53	0.850	0.000
	0.80	0.470	1.30	0.800	0.015	2.03	0.800	0.000
	0.75	0.525	1.47	0.750	0.019	2.17	0.750	0.000
	0.70	0.575	1.64	0.700	0.022	2.26	0.700	0.000
1.25	0.95	0.294	0.69	0.950	0.021	0.98	0.950	0.001
	0.90	0.415	1.98	0.900	0.042	1.59	0.900	0.002
	0.85	0.508	2.27	0.850	0.063	1.78	0.850	0.002
	0.80	0.587	2.20	0.800	0.084	2.02	0.800	0.003
	0.75	0.656	2.24	0.750	0.104	2.12	0.750	0.004
	0.70	0.719	2.26	0.700	0.125	2.19	0.700	0.005
1.5	0.95	0.352	2.50	0.950	0.053	0.95	0.950	0.006
	0.90	0.498	2.51	0.901	0.106	1.43	0.900	0.011
	0.85	0.610	2.65	0.851	0.159	1.94	0.850	0.017
	0.80	0.704	2.50	0.802	0.212	2.18	0.800	0.022
	0.75	0.788	2.42	0.752	0.266	2.61	0.750	0.028
	0.70	0.863	2.45	0.702	0.319	2.74	0.700	0.034

**Table 3:** Metrics summary for 16 MeV at 103 cm source‐to‐surface distance (SSD) at y = 0 cm 20 × 20 cm^2^ half‐modulated field for z = 0.5 cm (middle columns) and z = 2.0 cm (right columns).

r (cm)	IRF	d (cm)	z = 0.5 cm			z = 2.0 cm		
			d_T_	I_avg_	ΔI_R_	d_T_	I_avg_	ΔI_R_
0.5	0.95	0.117	0.56	0.950	0.042	0.63	0.950	0.000
	0.90	0.166	0.61	0.900	0.007	1.03	0.900	0.000
	0.85	0.203	0.94	0.854	0.001	1.22	0.851	0.000
	0.80	0.235	1.00	0.800	0.000	1.42	0.800	0.000
	0.75	0.263	1.00	0.749	0.001	1.42	0.749	0.000
	0.70	0.288	1.00	0.699	0.001	1.42	0.699	0.000
0.75	0.95	0.176	0.44	0.950	0.024	0.68	0.950	0.000
	0.90	0.249	0.73	0.900	0.031	1.03	0.900	0.001
	0.85	0.305	1.38	0.850	0.033	1.22	0.850	0.001
	0.80	0.352	1.55	0.800	0.044	1.25	0.800	0.001
	0.75	0.394	2.35	0.750	0.056	1.39	0.750	0.001
	0.70	0.431	1.90	0.700	0.067	1.48	0.701	0.002
1	0.95	0.235	1.17	0.950	0.056	0.59	0.950	0.007
	0.90	0.332	0.86	0.900	0.113	1.23	0.900	0.014
	0.85	0.407	1.54	0.850	0.169	1.37	0.850	0.021
	0.80	0.470	1.68	0.800	0.226	1.55	0.800	0.029
	0.75	0.525	1.50	0.751	0.282	1.90	0.750	0.036
	0.70	0.575	1.50	0.701	0.338	2.21	0.700	0.043
1.25	0.95	0.294	1.19	0.950	0.112	1.07	0.950	0.032
	0.90	0.415	1.29	0.901	0.243	2.09	0.900	0.064
	0.85	0.508	1.43	0.851	0.363	1.98	0.850	0.095
	0.80	0.587	1.50	0.801	0.485	2.10	0.800	0.127
	0.75	0.656	1.55	0.752	0.606	2.17	0.751	0.159
	0.70	0.719	1.56	0.702	0.728	2.17	0.700	0.191
1.5	0.95	0.352	0.67	0.951	0.191	2.20	0.951	0.071
	0.90	0.498	0.69	0.903	0.382	2.48	0.901	0.143
	0.85	0.610	0.97	0.854	0.573	2.40	0.852	0.214
	0.80	0.704	0.97	0.806	0.763	2.60	0.802	0.285
	0.75	0.788	0.99	0.757	0.955	2.54	0.752	0.357
	0.70	0.863	0.98	0.712	0.967	2.41	0.703	0.428

### 3.2 Evaluation of I_avg_

I_avg_ agrees within 0.001 of the intended values (0.70, 0.75, 0.80, 0.85, 0.90, and 0.95) for all combinations of beam energy, SSD, z, r, and IRF values, providing ∆I_R_ values are less than 0.10, which is well outside our acceptable criteria of 0.04. Hence, the I_avg_ acceptance criterion is redundant and unnecessary, as expected, as I_avg_ should equal the fraction of the beam unblocked by the island blocks. Furthermore, this reinforces the utility of passive intensity modulation using island blocks for IM‐BECT.

### 3.3 Evaluation of ∆I_R_

Ripple intensity trends smaller for lower energy, smaller block separation, and larger IRFs (nearer 1.0). A practical utilization of these data was to determine what are acceptable r values, that is, maximum block separation (r_max_), for a specific set of conditions, namely energy, SSD, and IRF_min_, the latter being the minimum value for a specific patient’s planned intensity distribution. As previously discussed, larger acceptable r values are more advantageous. Therefore, one preferred criterion might be to select the largest r value that keeps ∆I_R_ ≤ 4% at z = 2.0 cm. Data for z = 0.5 cm produce substantially greater ∆I_R_ values, which require progressively smaller r values if the shallow depth oscillations are in the patient, as opposed to being in the bolus. Further analysis of results is restricted to 103 cm SSD, since this is more typical of the clinic.

**Fig. 11:**
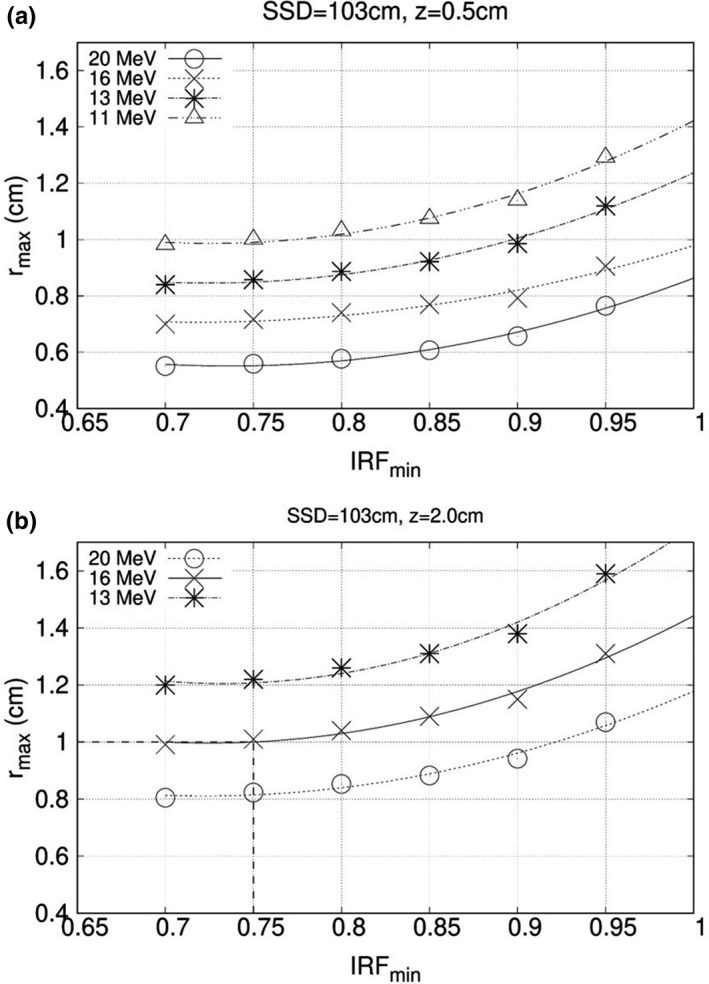
Maximum island block separation (r_max_) vs minimum IRF value in intensity‐modulated field (IRF_min_) for which ΔI_R_ is the maximum acceptable value, 4%. Plots are at specified beam energies (see inserted key) for 103 cm source‐to‐surface distance (SSD) and (a) depth z = 0.5 cm and (b) depth z = 2.0 cm, the latter corresponding to an average bolus thickness of 2 cm. For example, for conditions in (b) at 16 MeV and IRF_min_ value of 0.75 requires an island block separation less than 1.0 cm (as indicated by the dashed line). Curves are quadratic fits to data points.

These data are also useful for estimating the maximum island block separation for a given patient IM‐BECT geometry, which allows the fewest number of island blocks and the largest island block diameters, minimizing cost and effect of electron scatter into and from the island blocks. First, plots of ∆I_R_ vs r (cm) for multiple combinations of energies (11, 13, 16, and 20 MeV) and IRF values (0.70, 0.80, and 0.90) can be made for each of the four possible SSD and depths combinations. From these plots, the maximum island block separation (r_max_) can be extracted and plotted vs the IRF. Resulting plots are exemplified in Fig. 11 for the highest three energies (13, 16, and 20 MeV). Using these plots, based on beam energy and IRF_min_, a maximum island block separation value (r_max_) can be selected for use with a specific patient. For example, for the 16 MeV beam, 103 cm SSD, and 2.0 cm depth, an IRF_min_ of 0.85 has an r_max_ of 1.09 cm. It should be understood that the island block separation (r) can be decreased from r_max_ as needed for desired uniformity in individual patient plans.

### 3.4 Evaluation of d_T_

Though d_T_ had no formal pass/fail limit, for clinical use, the smallest d_T_ is preferred because it is a measure of how rapidly intensity could be modulated. Table 4 summarizes d_T_ values for a representative subset of all studied geometries at 103 cm SSD. From these results, it can be concluded that d_T_ trends smaller for higher energy, smaller SSD, shallower depth, just as penumbra widths at beam edges trend. Also, d_T_ trends smaller for IRF values closer to 1.0, simply a result of a gradient changing less over a shorter distance.

**Table 4:** Distance of Transition (d_T_) in cm for half‐beam intensity modulators (cf Fig. 3) in water at 103 cm source‐to‐surface distance (SSD) and depths of 0.5 cm (left) and 2.0 cm (right).

SSD = 103 cm Depth = 0.5 cm				SSD = 103 cm Depth = 2.0 cm			
7 MeV				7 MeV			
IRF	r = 0.5cm	r = 1.0cm	r = 1.5cm	IRF	r = 0.5cm	r = 1.0cm	r = 1.5cm
0.90	**1.49**	**1.56**	**1.33**	0.90	**2.19**	**2.18**	**2.12**
0.80	**1.90**	**1.95**	**2.61**	0.80	**2.85**	**2.84**	**2.77**
0.70	**2.26**	**2 .18**	2.98	0.70	**3.24**	**3.22**	**3.11**

13 MeV				13 MeV			
IRF	r = 0.5cm	r = 1.0cm	r = 1.5cm	IRF	r = 0.5cm	r = 1.0cm	r = 1.5cm
0.90	**1.00**	1.68	2.00	0.90	**1.26**	**1.24**	2.50
0.80	**1.05**	1.82	1.86	0.80	**1.65**	**1.58**	2.70
0.70	**1.26**	1.85	1.82	0.70	**1.83**	**1.75**	2.70

20 MeV				20 MeV			
IRF	r = 0.5cm	r = 1.0cm	r = 1.5cm	IRF	r = 0.5cm	r = 1.0cm	r = 1.5cm
0.90	**0.64**	0.92	0.88	0.90	**0.89**	1.27	1.79
0.80	**0.73**	1.06	1.13	0.80	**1.00**	1.54	1.75
0.70	**0.89**	1.21	0.74	0.70	**1.20**	1.61	1.66

Tables show data at beam energies of 7 MeV (top), 13 MeV (middle), and 20 MeV (bottom). d_T_ is shown for combinations of three IRF values (0.70, 0.80, and 0.90) and three hexagonal island block separations (0.5, 1.0, and 1.5 cm). d_T_ values have an estimated error of 0.05 cm. Clinically viable combinations (ΔI_R_ ≤ 4%) (cf Tables 5, 6) are bolded for clarity.

**Fig. 12:**
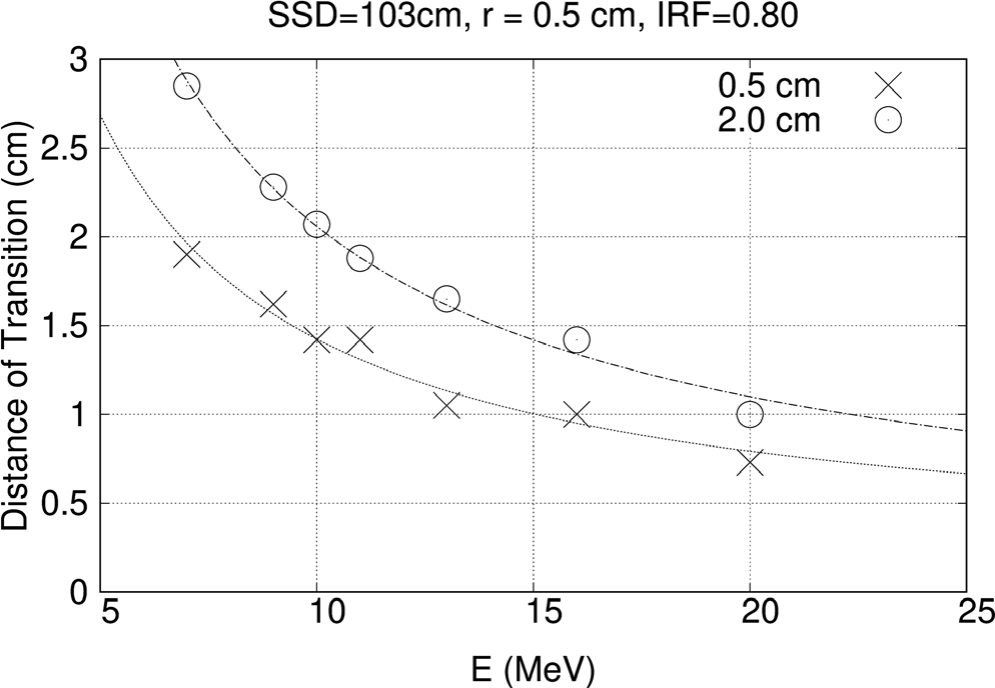
Distance of transition (d_T_) vs beam energy (E) and depth for 103 cm source‐to‐surface distance (SSD), r = 0.5 cm, and IRF = 0.80. Data at depth 0.5 cm (Xs); data at depth 2.0 cm (circles). For a given IRF and at both depths, d_T_ monotonically decreased with increased energy. Curves have been fit with a 1/E dependence.

Distance of transition monotonically decreased with energy, following an approximately 1/E dependence, similar to that of σ_θx_. This is illustrated by Fig. 12, which plots results at z = 0.5 and 2.0 cm for r = 0.5 cm and IRF = 0.80. The values at z = 0.5 cm are about 70% of those of z = 2.0 cm.

Not surprisingly, d_T_ was approximately constant with variation in r (0.5–1.5 cm), so long as ∆I_R_ (variation in IRF) was less than 2%. For example, for an IRF of 0.80 at 11 MeV and for z = 0.5 cm and z = 2.0 cm, d_T_ remained within 0.1 cm of an average of 1.32 and 1.89 cm, respectively.

### 3.5 Combinations (r,d) suitable for clinical use

Table 5 summarizes the scoring results at 100 cm SSD for all combinations (E, r) at depths z = 0.5 cm and z = 2.0 cm for all IRF values (0.70‐0.95). Combinations with a check denote those passing both I_avg_ and ΔI_R_ criteria for all IRF (>0.70), which are acceptable for intensity modulator design. For partial passes, the minimum permissible IRF is given, and where no modulation (IRF ≥ 0.70) is possible, the combination is marked “not acceptable” (N/A).

**Table 5:** Range of intensity reduction factors (IRF ≥ 0.70) of half‐beam intensity modulators (cf Fig. 3) that meet acceptability criteria (ΔI_R_ ≤ 4%) at 100 cm source‐to‐surface distance (SSD) and depths in water of z = 0.5 cm (top) and z = 2.0 cm (bottom).

SSD = 100 cm Depth = 0.5 cm					
E (MeV)	r = 0.5 cm	r = 0.75 cm	r = 1.0 cm	r = 1.25 cm	r = 1.5 cm
7	✓	✓	0.80	0.95	N/A
9	✓	✓	0.95	N/A	N/A
10	✓	0.85	N/A	N/A	N/A
11	✓	0.95	N/A	N/A	N/A
13	✓	N/A	N/A	N/A	N/A
16	0.85	N/A	N/A	N/A	N/A
20	N/A	N/A	N/A	N/A	N/A

SSD = 100 cm Depth = 2.0 cm					
E (MeV)	r = 0.5 cm	r = 0.75 cm	r = 1.0 cm	r = 1.25 cm	r = 1.5 cm
7	✓	✓	✓	✓	✓
9	✓	✓	✓	✓	0.85
10	✓	✓	✓	0.75	0.95
11	✓	✓	✓	0.90	N/A
13	✓	✓	0.80	0.95	N/A
16	✓	✓	0.95	0.95	N/A
20	✓	0.90	N/A	N/A	N/A

Beam energies (E).

Hexagonal separation (r).

Check marks demarcate acceptability of all island blocks for IRF ≥ 0.70.

Fractional numbers indicate the smallest IRF studied that meets acceptability criteria.

N/A indicated no IRF ≥ 0.70 met acceptability criteria.

At shallow depths (z = 0.5 cm), the results show that beam energies above 13 MeV do not exhibit sufficient scatter to produce clinically acceptable intensity distributions for the entire range of IRFs under consideration. In particular, 20 MeV beams may not be used under any conditions, and 16 MeV is limited to a minimum IRF of 0.85. For a deeper matching depth (z = 2.0 cm), beam energies up to and including 20 MeV can be used.

Table 6 summarizes the scoring results at 103 cm SSD for all combinations (E, r) at depths z = 0.5 cm and z = 2.0 cm for all IRF (0.70‐0.95), respectively. At this more clinical SSD, beam energies from 7 to 20 MeV have acceptable geometries for all IRF. In general, the 103 cm SSD allows larger block diameters, which as previously mentioned, have advantages.

**Table 6:** Range of intensity reduction factors (IRF ≥ 0.70) of half‐beam intensity modulators (cf Fig. 3) that meet acceptability criteria (ΔI_R_ ≤ 4%) at 103 cm source‐to‐surface distance (SSD) and depths in water of z = 0.5 cm (top) and z = 2.0 cm (bottom).

SSD = 103 cm Depth = 0.5 cm					
E (MeV)	r = 0.5 cm	r = 0.75 cm	r = 1.0 cm	r = 1.25 cm	r = 1.5 cm
7	✓	✓	✓	✓	0.80
9	✓	✓	✓	0.85	0.95
10	✓	✓	✓	0.95	N/A
11	✓	✓	✓	0.95	N/A
13	✓	✓	0.95	N/A	N/A
16	✓	0.85	N/A	N/A	N/A
20	✓	0.95	N/A	N/A	N/A

SSD = 103 cm Depth = 2.0 cm					
E (MeV)	r = 0.5 cm	r = 0.75 cm	r = 1.0 cm	r = 1.25 cm	r = 1.5 cm
7	✓	✓	✓	✓	✓
9	✓	✓	✓	✓	✓
10	✓	✓	✓	✓	✓
11	✓	✓	✓	✓	0.85
13	✓	✓	✓	0.80	0.95
16	✓	✓	0.75	0.95	N/A
20	✓	✓	0.95	N/A	N/A

Beam energies (E).

Hexagonal separation (r).

Check marks demarcate acceptability of all island blocks for IRF ≥ 0.70.

Fractional numbers indicate the smallest IRF studied that meets acceptability criteria.

N/A indicated no IRF ≥ 0.70 met acceptability criteria.

## 4 SUMMARY AND CONCLUSION

The objective of this study was to determine combinations of block diameter and hexagonal grid separation, which could be used to produce clinically acceptable intensity distributions for IM‐BECT, while minimizing ∆I_R_ and d_T_. A pencil beam algorithm was used to calculate the relative fluence (intensity) distribution beneath a half‐modulated 20 × 20 cm^2^ field (island blocks on positive x‐axis) for a range of hexagonal separations (0.5–1.5 cm) and IRFs (0.70–0.95), having island block diameters (d = 0.117–0.863 cm) at depths of z = 0.5 cm and z = 2.0 cm. This was done both at 100 cm SSD (air gap = 5.0 cm) and at an extended 103 cm SSD (air gap = 8.0 cm) for beam energies of 7–20 MeV.

Results showed that (1) the average intensity agreed with the intended intensity within 0.001 as long as ∆I_R_ was within a clinically acceptable range (≤0.04) and (2) ∆I_R_ was clinically acceptable in limited regions of E, SSD, r, IRF, and z space. For example, the use of 20 MeV beams was precluded at 100 cm SSD and shallow depth (z = 0.5 cm), and the 16 MeV beam was limited to cases with IRF ≥ 0.85. However, using a more clinical 103 cm SSD, ∆I_R_ was acceptable for all energies (7‐20 MeV) and depths (z = 0.5 and 2.0 cm). Also, the data provided plots for specific conditions from which the maximum island block separation (r_max_) could be extracted.

Although selecting solutions with the largest block separation (r) and thus the largest diameter blocks may have some fabrication and block scatter advantages, this comes with the disadvantage of slightly increased distance of transition (d_T_), which could limit the gradient of sharply varying intensity‐modulating patterns. If necessary, these competing effects can be properly balanced in the planning process, which will depend on the wide range of data computed for this study.

We conclude that these data are useful in determining island block hexagonal separation and hence island block diameters required to produce electron beam intensity modulators for individual patients receiving IM‐BECT.
